# Monitoring swine virus transmission in embryos derived from commercial abattoir oocytes

**DOI:** 10.3389/fvets.2024.1336005

**Published:** 2024-02-02

**Authors:** Brent Pepin, Paula Rodriguez-Villamil, Lauren Sammel, Jie Yin, Brian Dacken

**Affiliations:** ^1^Cytotheryx, Inc., Rochester, MN, United States; ^2^Recombinetics, Inc., Eagan, MN, United States; ^3^Sustainable Swine Resources LLC, Watertown, WI, United States

**Keywords:** porcine circovirus, porcine cytomegalovirus, porcine endogenous retrovirus, SCNT, xenotransplantation, infectious disease, parthenogenic embryo, cloning

## Abstract

Pigs are pivotal in agriculture and biomedical research and hold promise for xenotransplantation. Specific-pathogen-free (SPF) herds are essential for commercial swine production and xenotransplantation research facilities. Commercial herds aim to safeguard animal health, welfare, and productivity, and research facilities require SPF status to protect immunocompromised patients. Somatic cell nuclear transfer (SCNT) embryos are the norm for producing cloned and genetically edited animals. Oocytes for embryo reconstruction are most conveniently sourced from commercial abattoirs with unclear disease statuses. However, research on viral clearance from donor oocytes during embryo reconstruction remains limited. SCNT has previously been shown to reduce the transmission of Porcine reproductive and respiratory syndrome virus, Bovine viral diarrhea virus, Porcine Circovirus type 2, and Porcine parvovirus. Still, it is lacking for other pathogens, including endogenous viruses. This project contains two preliminary studies investigating the polymerase chain reaction (PCR) assay detection of common swine viruses through the phases of producing parthenogenic and SCNT embryos. Exogenous pathogens detected in oocyte donor tissue or the oocyte maturation media were not detected in the produced embryos. Porcine endogenous retrovirus type C (PERVC) was not removed by parthenogenic embryo activation and was detected in 1 of the 2 tested SCNT embryos reconstructed using a PERVC-negative cell line. SCNT and parthenogenic embryo construction similarly reduced exogenous virus detection. SCNT embryo construction helped reduce endogenous virus detection. This project demonstrates the importance of screening embryos for endogenous viruses and shows the usefulness of parthenogenic embryos in future exogenous virus clearance studies.

## 1 Introduction

Maintaining a high health status in commercial pork production is vital for enhancing animal welfare, optimizing feed utilization, reducing production costs, and minimizing antibiotic usage (1–3). As gene-editing technology advances, there is growing interest in SCNT coupled with gene editing to produce disease-resistant or higher producing animals (1–3). This technology also offers a means to preserve valuable animal genetics (4–7). Genetically modified pigs hold promise for xenotransplantation by reducing the organ rejection rate and facilitating the development of biomedical models (4–7). However, using swine organs in transplantation risks zoonotic disease transmission to immunosuppressed hosts, even with pathogens not typically associated with human disease (8–11).

Raising pigs in SPF facilities and using SCNT to produce pigs have been shown to minimize pathogen exposure (9–11). However, commercial abattoirs are the common source for oocyte collection, and they receive pigs of unknown disease statuses, posing a risk of exposure to SPF facilities through oocyte contamination or infection (10, 12). SCNT has demonstrated reduced Porcine reproductive and respiratory syndrome virus (PRRSV), Bovine viral diarrhea virus (BVDV), Porcine Circovirus type 2 (PCV2), and Porcine parvovirus (PPV) transmission when oocytes are adequately washed after collection, but data on other viruses is limited (10, 12, 13).

Swine viruses cited as a possible concern for xenotransplantation include Porcine Circovirus type 1 (PCV1), PCV2, Porcine circovirus type 3 (PCV3), Porcine cytomegalovirus (PCMV), Swine Hepatitis E virus (HEV), Porcine hemagglutinating encephalomyelitis virus (PHEV), Porcine lymphotropic herpesvirus 1, 2, and 3 (PLH1, PLH2, PLH3), and PERVC (12, 14–16). Additionally, other viruses, while potentially not relevant to xenotransplantation, can impact the health and production of SPF facilities include Influenza A virus (IAV), PPV, Porcine endemic diarrhea virus (PEDV), Transmissible gastroenteritis virus (TGEV), Porcine delta coronavirus (PDCoV), Porcine rotavirus A, B, and C (ROTA, ROTB, ROTC), and Seneca Valley Virus (SVV).

Parthenogenic embryos can increase the number of embryos for evaluation without completing the complicated SCNT reconstruction process (17, 18). Parthenogenic embryos are unfertilized oocytes activated into embryos that cannot progress beyond the early developmental stages and do not produce full-term pregnancies (17, 18). Parthenogenic embryos may allow a more accessible alternative to SCNT cloning when screening the potential viral risk of oocytes to embryo reconstruction by increasing the number of embryos available for diagnostic testing.

Current standards for oocyte or embryo testing for SCNT cloning within SPF swine herds require development and agreement within the industry. This project aims to expand industry knowledge by piloting parthenogenic embryos as a screening method for viruses of concern. Furthermore, the project seeks to trace both exogenous and endogenous viruses from oocyte collection through embryo reconstruction.

This project comprises two preliminary studies designed to evaluate potential testing points and assess the risk of disease transmission from oocytes collected from commercial abattoirs to reconstructed embryos. Study 1 focuses on the activation of parthenogenetic embryos to determine their suitability for virus clearance studies, while study 2 uses SCNT-cloned embryos, representative of the cloning process. Both studies employ PCR assays to detect viral swine pathogens at various stages of oocyte collection to embryo formation, intending to mitigate potential risks to xenotransplantation receipts and SPF herd production.

## 2 Materials and methods

### 2.1 Media preparation

Maturation media A (MMA) and maturation media B (MMB) stock solutions were prepared following previously described methods (19). In brief, MMA is formed by combining 500 mL of Medium 199 (MilliporeSigma, St. Louis, MO, United States) with 0.5 g polyvinyl alcohol, 0.15 g sodium bicarbonate, and 10 μg/mL gentamicin. MMB is prepared by combining 100 mL of MMA, 0.055 g of glucose, and 0.001 g of sodium pyruvate (19).

Oocyte maturation medium (OMM) was formed from a stock solution of MMB using previously described methods (19). In brief, 40 mL of MMB is combined with 10 ng/mL epidermal growth factor, 0.5 μg/mL luteinizing hormone, 0.5 μg/mL follicle stimulating hormone, 0.57 mM L-cysteine, 40 ng/mL fibroblast growth factor 2, and 20 ng/mL leukemia inhibitor factor then incubated at 38.5°C.

Holding media and wash media was a HEPES solution (MilliporeSigma, St. Louis, MO, United States) modified using previously described methods (19). In brief, 2.383 g HEPES solution was combined with 6.663 g NaCl, 0.239 g KCl, 0.168 g NaHCO_3_, 0.041 g NaH_2_PO_4_, 1.868 g sodium lactate, 0.102 g MgCl_2_·2H_2_O, 0.294 g CaCl_2_·2H_2_O, 0.1 g polyvinyl alcohol, 2.186 g sorbitol, 0.022 g sodium pyruvate, 10 μg/mL gentamicin, and 0.01 g phenol red followed by filtering through 0.22 μM filter (19).

### 2.2 Oocyte collection process

Oocytes were collected and prepared from abattoirs in Abbyland and Watertown, Wisconsin, from the ovaries derived from sows after euthanasia at a local abattoir under standard processing procedures. Briefly, freshly obtained ovaries were transported to the onsite laboratory facility and immediately washed in warmed physiological saline containing 10 μg/mL gentamicin. Ovarian follicles (3–5 mm in diameter) were aspirated with an 18-gauge needle attached to a 10 mL syringe. The aspirates were placed into a 50 mL conical tube and allowed to settle in a 38.5°C water bath. After discarding the follicular fluid supernatant, the pellet was resuspended 1:1 (v/v) in holding media for washing. This washing step was repeated three times before transferring the clean follicular fluid to a 100 mm petri dish and searched by a dissecting microscope. Oocytes with a homogenous-colored cytoplasm and at least two cumulus cell layers were transferred by a denudation and handling pipettor (CooperSurgical, Inc., Trumbull, CT) to a 35 mm petri dish of holding media before being placed in OMM equilibrated to 38.5°C and 5% CO_2_. Cumulus oocyte complexes were transferred into 1.5 mL tubes containing an equilibrated OMM. The tubes were sealed with parafilm before being shipped overnight to Recombinetics, Inc. in Eagan, Minnesota, in a transport incubator that can hold all the tubes at a constant 38.5°C.

### 2.3 Study 1: parthenogenic embryos

Study 1 screened for multiple pathogens in OMM, donor oocytes, and the activated parthenogenic embryos of those oocytes. Pathogens screened for were BVDV, PRRSV, PCV1, PCV2, PCV3, PPV, PCMV, PDCoV, TGEV, PEDV, SVV, HEV, IAV, PERVC, PHEV, PLH1, PLH2, PLH3, ROTA, ROTB, and ROTC. These pathogens were selected given their prevalence in commercial swine herds or, in the case of BVDV, the concern for xenotransplantation research.

The oocytes (*n* = 300) were transported at 38.5°C in OMM (OMM-1 for sampling). After 24 h, the oocytes were washed and transferred to 500 μL wells of fresh OMM (OMM-2 for sampling) for 18 h. After use, a 3 mL sample of OMM-1 and OMM-2 was collected for diagnostic testing.

After 18 h, 60 oocytes were separated and submitted for diagnostic testing, and the rest of the matured oocytes (*n* = 200) were chemically activated, as previously described, to produce parthenogenic embryos (17). In brief, oocytes were exposed to 5 μM ionomycin HEPES-buffered medium supplemented with bovine serum albumin and incubated for 4 h, followed by a culture in PZM-3 medium for 7 days (17). After a 7-day evaluation, 150 parthenogenic embryos were collected and submitted for pathogen screening. All samples were submitted to the University of Minnesota Veterinary Diagnostic Laboratory, a fully accredited American Association of Veterinary Laboratory Diagnosticians lab, and tested by PCR diagnostic assays according to the laboratory’s standard diagnostic procedures using the laboratory’s provided cycle threshold (ct) cut-offs. An IAV ct <35 was considered positive. PHEV was positive with ct <36, suspect at ≥36 but <45. PRRSV, PEDV, TGEV, and PDCoV were positive at ct <40. PLHVs were assayed using standard gel electrophoresis PCR with only positive or negative results. All other viruses were positive with ct <36, suspect at ≥36 but <40.

### 2.4 Study 2: SCNT-produced embryos

Study 2 tested PCV2, PCV3, PCMV, and PERVC by PCR assays throughout the oocyte collection process to SCNT reconstructed embryos. PCR diagnostics were performed on the donor sow tissue, accumulated ovarian fluid during collection, the follicular fluid from the ovaries, the oocytes, the DNA donor cell line for cloning with the SCNT procedure, and the reconstructed embryos. Approximately 1 cm^3^-sized lung and spleen samples were collected from the same animals from which ovaries were procured for obtaining oocytes. The lung and spleen samples were pooled daily over 3 days (75–100 sows per day with 275 sows total represented). The lung and spleen samples were homogenized using a standard blender with equal cold saline. Five 5 mL samples were collected daily (5 oocyte donor tissue samples per day, 15 samples total). The ovaries from each sow were compiled into a clean, separate container. Five 5 mL samples of the fluid accumulated in the collection container for the ovaries were submitted for diagnostic testing (5 accumulated ovarian fluid samples per day, 15 samples total). Follicular fluid was collected by removing 10 2 mL samples from the top of follicular fluid tubes (10 follicular fluid samples per day, 30 samples total). After the enucleation and washing, as previously described, five pools of 20 oocytes each were collected (5 oocyte pools per day, 15 samples total) (18). The donor cell lines for SCNT embryo reconstruction were submitted for diagnostic testing. Of the completed SCNT embryos produced as previously described, 40 were separated on the first 2 days of sampling for diagnostic testing (2 samples total) (18). The pathogens tested were selected given the expected prevalence of these diseases in commercial swine herds and their relevance to xenotransplant research. Lung and spleen from donor animals were chosen due to their ease of collection on an abattoir production line and their documented harboring of the selected pathogens in this study.

All samples were submitted to Iowa State University Veterinary Diagnostic Laboratory, a fully accredited lab by the American Association of Veterinary Laboratory Diagnosticians, for DNA extraction. Iowa State University Veterinary Diagnostic Laboratory tested each sample by PCR for PCV2, PCV3, and PCMV. Extracted DNA samples were then sent to the University of Minnesota Veterinary Diagnostic Laboratory for PERVC PCR testing. All DNA extraction and diagnostic testing were performed according to the laboratory’s standard diagnostic procedures. PCMV was considered positive with ct < 35, and PCVs were positive with ct < 37 at Iowa State University Veterinary Diagnostic Laboratory.

## 3 Results

### 3.1 Study 1

As seen in Table 1, BVDV, PCMV, and PERVC were detected at positive or suspect ct values in the OMM1 and OMM2. Oocytes were positive for BVDV and detected at suspect levels for SVV. Oocytes could not be tested for nine viruses due to insufficient sample presence to reserve enough oocytes to produce parthenogenic embryos to test for all pathogens. Embryos were positive for PERVC but negative for all other viruses.

**Table 1:** Pathogens detected in the process of parthenogenic embryo construction.

Sample type tested (*n* = 1 for each sample)	Pathogens detected by sample type tested^†^			
	BVDV	PCMV	SVV	PERVC
Oocyte maturation media 1	Negative	Suspect	Negative	Positive
Oocyte maturation media 2	Positive	Suspect	Negative	Positive
Oocytes	Positive	Negative	Suspect	Not Tested
Parthenogenic embryos	Negative	Negative	Negative	Positive

^†^All testing was performed by PCR for the pathogens at an accredited diagnostic laboratory according to standard diagnostic procedures. Pathogens that tested negative on all tested samples and were not included in the table are PRRSV, PCV1, PCV2, PCV3, PPV, PDCoV, TGE, PEDV, HEV, IAV, PHEV, PLH1, PLH2, PLH3, ROTA, ROTB, and ROTC. HEV, IAV, PHEV, PLH1, PLH2, PLH3, ROTA, ROTB, and ROTC were not tested on oocytes due to ensuring enough oocytes were available to produce enough parthenogenic embryos for testing of all pathogens on that sample type.

### 3.2 Study 2

As seen in Table 2, PCMV was negative on all samples. PCV2 and PCV3 were detected in all donor sow tissue samples and 53–80% of accumulated ovarian fluid samples. PCV2 and PCV3 were negative in all follicular fluid, oocytes, and reconstructed embryos. PERVC was detected in 100% of the samples of donor sow tissue, accumulated ovarian fluid, follicular fluid, and oocytes. The donor cell line for SCNT embryo reconstruction was negative for PERVC on all tests.

**Table 2:** Pathogen detection from stages of oocyte collection to SCNT embryo reconstruction.

Sample type tested	PCMV	PCV2	PCV3	PERVC
	positive/total (% positive)^†^	Positive/total (% positive)	Positive/total (% positive)	Positive/total (% positive)
Oocyte donor tissues^‡^	0/15 (0)	15/15 (100)	15/15 (100)	15/15 (100)
Accumulated ovarian fluid^§^	0/15 (0)	8/15 (53)	12/15 (80)	15/15 (100)
Follicular fluid	0/30 (0)	0/30 (0)	0/30 (0)	30/30 (100)
Oocytes	0/15 (0)	0/15 (0)	0/15 (0)	15/15 (100)
Donor cell line^¶^	0/2 (0)	0/2 (0)	0/2 (0)	0/2 (0)
Reconstructed embryos^††^	0/2 (0)	0/2 (0)	0/2 (0)	1/2 (50)

^†^All testing was performed by PCR for the pathogens at accredited diagnostic laboratories on extracted DNA according to standard diagnostic procedures. Column depicts the number of positive samples/total samples tested (% positive samples). ^‡^Oocyte donor tissue was a homogenate of pooled lung and spleen samples from each row that had ovaries collected for oocyte harvesting. ^§^Ovary container fluid samples consisted of the residual fluid accumulated at the bottom of the container into which donor sow ovaries were collected. ^¶^The same cell line was used for all embryo reconstruction and was tested twice. ^††^Two samples of 40 embryos each were separated from the reconstructed embryos for pathogen PCR testing.

## 4 Discussion

To the authors’ knowledge, this project is the first to attempt the detection of BVDV, PCV1, PCV3, PCMV, IAV, PEDV, TGEV, PDCoV, ROTA, ROTB, ROTC, and PERVC at various stages, from abattoir oocyte collection through embryo parthenogenesis or SCNT embryo formation in swine. Previous work has demonstrated a reduction in the transmission of PRRSV, BVDV, PCV2, and PPV when oocytes are adequately washed after collection for SCNT but did not explore the potential use of parthenogenetic embryos through reconstruction for pathogen testing (10, 12, 13).

Preliminary study 1 revealed the potential to detect BVDV and PCMV in OMM (Table 1). Cattle are BVDV’s natural host; however, the prevalence of BVDV in commercial pig herds has been demonstrated to varying degrees (20). A low risk of transferring BVDV to bovine SCNT embryos was previously shown but not examined in swine (13). Our study suggests that BVDV transfer is also a low risk in swine embryo reconstructions but needs confirmation with swine SCNT-constructed embryos. PCMV is highly prevalent in commercial herds and detectable by various sample types, making its detection expected (21). SVV was detected at suspect ct levels from the oocytes but not in the media. The possibility of the vertical transmission of SVV has been suggested but not confirmed, making the interpretation of finding SVV in the oocytes but not the media unclear (22). It is possible that SVV in the media was too dilute to provide a suspect or positive detection but was at a greater concentration in the direct oocyte sample.

Notably, BVDV, PCMV, and SVV were not detected in the parthenogenic embryos, supporting evidence that the oocyte washing and embryo formation process helps reduce the risk of exogenous virus transfer. However, this needs to be repeated in a robust study for confirmation as the sample size was very limited in this preliminary proof of concept study. The absence of detection for the other study 1 pathogens likely reflects a sampling of negative animals, prevention of fecal contamination, or tissue tropism for those pathogens. This preliminary proof of concept study highlights the potential clearance of exogenous viruses, underscoring the use of parthenogenic embryos for cost savings and increased embryo sample size for diagnostics in future viral clearance studies.

Preliminary study 2 displayed a high detection rate of PCV2 and PCV3 in oocyte donor tissues and the accumulated ovarian fluid (Table 2). The high detection rates are expected, given the high prevalence of the viruses in commercial herds (23). PCV2 and PCV3 are detectable by PCR in the serum and various tissues and have been known to infect fetuses (23, 24). Contrary to a previous study that found PCV2-infected oocytes and infected SCNT embryos, our study did not detect PCV2 or PCV3 in follicular fluid, oocytes, or reconstructed embryos (25). This discrepancy may be due to regional strain differences, as the previous study used a nonpathogenic strain from a Chinese abattoir vs. the United States strain in the current study (25). Potential differences in oocyte decontamination and SCNT reconstruction practices may also contribute to differences in PCV2 detection in the embryos and oocytes (13). In study 2, PCMV was not detected in the donor sows, preventing the confirmation of study 1, which detected PCMV in the maturation media. The lack of detection of PCMV in the donor sows was unexpected since it is well-documented as a common virus in domestic pigs and can be detected in both spleen and lung samples by PCR (21). However, PCMV, like other herpesviruses, can enter a latent stage, especially in adult animals, making detection in tissue more difficult due to the viral load being below detection limits (21). PCMV has also been shown to vary in distribution in individual animals (21). Further research on the disease transmission of PCMV from oocytes collected from positive sows to reconstructed SCNT embryos is needed. In future studies, anti-PCMV antibodies or more varied organ testing may be warranted to confirm the presence in donor animals.

As for PERVC, it is widely distributed in the genomes of pigs (26). In study 1, the nucleus is not removed from the oocytes for the development of parthenogenic embryos, which means a PERVC-positive oocyte would remain a PERVC-positive embryo. In the production of SCNT embryos, removing the oocyte nucleus is necessary (13). Nucleus removal from a PERVC-positive oocyte would be expected to provide a PERVC-negative embryo if the donor cell line used for SCNT reconstructions was PERVC-negative. However, in study 2, only 1 of the 2 tested SCNT embryos were PERVC-negative after replacing the oocyte nucleus with a PERVC-negative donor cell line. Incomplete oocyte enucleation is the probable reason for the PERVC-positive embryo (27). This study emphasizes the importance of testing embryos or resulting piglets to ensure successful pathogen elimination for viruses capable of genome integration. Limitations of the current project include the small sample size, the need for more rigorous sampling, the shortage of oocytes for completing all testing in study 1, and the inability to confirm the PCMV status of donor animals in study 2.

## 5 Conclusion

In conclusion, this project explores two different embryo construction methods and the detection of viruses at various construction steps to analyze pathogen elimination in the final embryo product. The pathogens requiring monitoring will likely vary across specific swine herds, facilities, pig tissue of interest, and production goals. Except for PERVC, viruses detected in the early process stages were not seen in the final product embryos. These studies suggest that embryos constructed from oocytes collected at commercial abattoirs have a low disease risk for transferring exogenous viruses when adequately washed. The sample size and replicates in both studies are too small to draw hard conclusions, but this apparent minimal risk aligns with previous studies on select exogenous viruses (10, 12, 13). Future studies on the clearance of exogenous viruses in embryo reconstruction may benefit from using parthenogenetic embryos to increase available material for testing without the complications of SCNT. Using parthenogenic embryos from study 1 for diagnostics still requires confirmation in a more extensive, robust study with appropriate replicates.

For exogenous viral pathogens, this project suggests that proper oocyte handling for SCNT and parthenogenic embryo construction assist in reducing the potential risk of viral transmission by embryo transfer. This potential risk reduction is encouraging for using SCNT in SPF herds to lower the threat of zoonotic diseases in xenotransplantation research herds and for pathogens that may significantly impact animal production in commercial herds. However, it emphasizes that for endogenous viruses like PERVC, testing the embryos or resulting piglets may be recommended to confirm that viral transfer to offspring did not occur. Even with a negative donor cell line, incomplete oocyte enucleation may lead to an endogenous virus-positive embryo.

These small preliminary studies underscore the need for more extensive investigations with more robust sample collection methods for pathogen clearance from oocyte collection through embryo reconstruction, especially for endogenous pathogens. The small sample sizes and the lack of confirmation of infection in donor animals for PCMV limit the interpretation and prevent statistical analysis of study results.

## Data availability statement

The original contributions presented in the study are included in the article/supplementary material, further inquiries can be directed to the corresponding author.

## Ethics statement

Ethical approval was not required for the studies involving animals in accordance with the local legislation and institutional requirements because this study did not involve using any live animals for completion, and the product was only the creation of embryos. Samples were collected from animals already euthanized at a commercial abattoir under the facility's current standard processing practices. The facility regularly collects samples, like oocytes, as part of its standard practices. No live animals were harmed or used in the completion of this project, and no animals were euthanized specifically for this project. Written informed consent was not obtained from the owners for the participation of their animals in this study because the animals were at the point of collection under the ownership of Sustainable Swine Resources, who are contributing authors to this paper, and they were the ones who collected the oocyte and tissue samples from the already euthanized animals in their abattoir.

## Author contributions

BP: Conceptualization, Data curation, Investigation, Methodology, Project administration, Supervision, Writing – original draft, Writing – review & editing. PR-V: Conceptualization, Data curation, Investigation, Methodology, Project administration, Supervision, Writing – review & editing. LS: Data curation, Investigation, Methodology, Writing – review & editing. JY: Data curation, Investigation, Methodology, Writing – review & editing. BD: Conceptualization, Methodology, Writing – review & editing.

## Figures and Tables

**Table:** 

BVDV	Bovine viral diarrhea virus
CT	cycle threshold
HEV	Swine hepatitis E virus
IAV	Influenza A virus
MMA	Maturation media A
MMB	Maturation media B
OMM	Oocyte maturation medium
PCMV	Porcine cytomegalovirus
PCR	Polymerase chain reaction
PCV1	Porcine circovirus type 1
PCV2	Porcine circovirus type 2
PCV3	Porcine circovirus type 3
PDCoV	Porcine delta coronavirus
PEDV	Porcine endemic diarrhea virus
PERVC	Porcine endogenous retrovirus type C
PHEV	Porcine hemagglutinating encephalomyelitis virus
PLH1	Porcine lymphotropic herpesvirus 1
PLH2	Porcine lymphotropic herpesvirus 2
PLH3	Porcine lymphotropic herpesvirus 3
PPV	Porcine parvovirus
PRRSV	Porcine reproductive and respiratory syndrome virus
ROTA	Porcine rotavirus A
ROTB	Porcine rotavirus B
ROTC	Porcine rotavirus C
SCNT	somatic cell nuclear transfer
SPF	Specific-pathogen-free
SVV	Seneca Valley Virus
TGEV	Transmissible gastroenteritis virus
